# Effects of lipoteichoic and arachidonic acids on the immune-regulatory mechanism of bovine mammary epithelial cells using multi-omics analysis

**DOI:** 10.3389/fvets.2022.984607

**Published:** 2022-08-24

**Authors:** Weitao Dong, Yan Chen, Quanwei Zhang, Xiaoxuan Zhao, Peiwen Liu, Haijian He, Ting Lu, Yuxuan He, Xianghong Du, Junjie Hu, Xingxu Zhao, Yong Zhang

**Affiliations:** ^1^College of Veterinary Medicine, Gansu Agricultural University, Lanzhou, China; ^2^Key Laboratory of Animal Reproductive Physiology and Reproductive Regulation of Gansu Province, Lanzhou, China; ^3^College of Life Science and Technology, Gansu Agricultural University, Lanzhou, China

**Keywords:** *Staphylococcus aureus*, lipoteichoic acid, arachidonic acid, proteomics, transcriptomics, metabolomics

## Abstract

*Staphylococcus aureus* is one of the most important pathogens causing mastitis in dairy cows. It mainly utilizes the properties of its pathogenic factor, lipoteichoic acid (LTA), to elicit a host-cell inflammatory response and evade the host-cell immune response. Arachidonic acid (AA) has a regulatory role in the inflammatory response, cell metabolism, and apoptosis. The study aimed to establish a cell model by determining the optimal concentration of LTA and AA for cell induction using the Cell Counting Kit−8 assay and the quantitative polymerase chain reaction of *interleukin* (*IL*)*-1*β, *IL-2*, and *IL-6*. MAC-T cells were planted in 36 10-cm^2^ culture dishes at a density of 1 × 10^7^ cells per dish. They were treated with LTA for 24 h to constitute the LTA group and with AA for 12 h to constitute the AA group. The cells were pretreated with LTA for 24 h followed by treatment with AA for 12 h to constitute the LTA + AA group. Using proteomic, transcriptomic, and metabolomic analyses, this study determined that LTA can regulate the expression of *Actin Related protein 2/3 complex* (*ARPC*)*3, ARPC4, Charged Multivesicular Body Protein 3, protein kinase cGMP-dependent, NF-*κ*B Inhibitor Alpha*,and other genes to affect cellular metabolism, immune regulation and promote apoptosis. In contrast, AA was observed to regulate the expression of genes such as *ARPC3, ARPC4, Charged Multivesicular Body Protein 3, Laminin Gamma 1, Insulin Receptor, Filamin B*, and *Casein Kinase 1 Epsilon* to inhibit cellular apoptosis and promote immune regulation, which provides a theoretical basis for future studies.

## Introduction

Mastitis is one of the dairy cow diseases that is widespread, difficult to control, and expensive to treat worldwide (1). It can reduce the milk production and raw milk quality in dairy cows and increase the cost of treatment and culling of sick cows, causing huge economic losses in the dairy industry (2). *Staphylococcus aureus* (*S. aureus*) is one of the main pathogens causing mastitis in dairy cows. In contrast to acute inflammation caused by *Escherichia coli* (*E. coli*) infection of the mammary gland, *S. aureus* causes a delayed inflammatory response in cows, mainly in the form of low concentrations of neutrophil chemo-attractants [*interleukin* (*IL*)-8, *complement component 5a* (*C5a*)] and *neutrophil potent activators* [*tumor necrosis factor* (*TNF)-*α] in milk (3). Therefore, it can be inferred that the cytokine environment induced by *S. aureus* infection in the host may not be the optimal environment for neutrophils to reach their highest bactericidal potential, owing to which this pathogen appears to be persistently infected intracellularly (4). Additionally, when the surrounding environment is not conducive to its survival, *S. aureus* forms a dense biofilm that makes it more resistant to antibiotics to reproduce after the environment is restored (5). Therefore, the culling rate of cows with mastitis owing to *S. aureus* infection increases (6).

Lipoteichoic acid (LTA) is a unique alditol polymer in the cell wall of gram-positive bacteria, which is attached to the membrane mainly by lipid anchors (7). Similar to lipopolysaccharide (LPS) in gram-negative bacteria, LTA is a pathogen-associated molecular pattern in gram-positive pathogenic bacteria and plays an important role in bacterial-host cell interactions, inflammatory response, and regulation of immune response (8). It also plays an important role in the pathogenesis of *S. aureus* and helps it adhere to and colonize host cells to stimulate their inflammatory response (9). It induces the production of inflammatory mediators in monocytes and macrophages, including *TNF-*α, *leukotriene B4, IL-1*β, *IL-2, IL-6, C5a, monocyte chemotactic protein-1* (*MCP-1*), and *macrophage inflammatory protein-1*α (*MIP-1*α) (10). Massive neutrophil recruitment and increased cytokines and chemokines are hallmarks of LTA-induced inflammation (11).

There is a positive regulatory relationship between oxidative stress and inflammatory response. Arachidonic acid (AA) has a bidirectional regulatory effect on oxidative stress. AA generates large amounts of reactive oxygen species (ROS) to stimulate the oxidative stress response. For example, AA can activate calcium channels in the cell membrane, thus, increasing the concentration of calcium ions in the calcium cell, which activates nicotinamide adenine dinucleotide phosphate oxidase and generates large amounts of ROS (12). AA can also inhibit oxidative stress by increasing antioxidant enzyme activity. For example, AA increases the activity of four antioxidant enzymes, including Cu, Zn superoxide dismutase, manganese superoxide dismutase, glutathione peroxidase, and catalase, by activating peroxisome proliferator-activated receptor (13–15).

In summary, this study aimed to construct a biological model of mastitis caused by *S. aureus* through LTA-induced immortalized bovine mammary epithelial (MAC-T) cells. We also added exogenous AA to the system and used multi-omics to investigate the mechanism of AA regulation on the inflammatory response induced by LTA and to screen for potential target genes and metabolites.

## Materials and methods

### Cell line and induced concentration screening

Professor Xiao LongFei at the Beijing University of Agriculture donated MAC-T cells. To cultivate the cells, we utilized Dulbecco's Modified Eagle Medium/HIGH GLUCOSE media (Hyclone, SH300, Tauranga, New Zealand) supplemented with 15% fetal bovine serum (FBS) (Invigentech, A6901FBS-500, California, USA) and incubated them at 37°C in 5% CO_2_ incubator. The cells were digested and passaged with 0.02% ethylenediamine tetraacetic acid and 0.25% trypsin. Cells were expanded and cultured to the 10th generation for subsequent experiments.

MAC-T cells were inoculated in 96-well plates at 1 × 10^4^ per well, randomly divided into six groups of five replicates each, and 300 μL of complete medium was added to each well, and 300 μL of Phosphate-Buffered Saline (PBS) was added to each well at the edge (16). In 12 h specific incubation period, five cells were randomly selected as the control group, the old medium was removed, 200 μL of complete medium was added, and the remaining columns were replaced with 200 μL of a complete medium at different concentrations of LTA (0.1, 1, 5, 10, 20, or 40 μg/mL). According to the instructions of the Cell Counting Kit-8 (CCK-8) kit, each well-received a 10-μL CCK-8 solution and was incubated for 2 h at 37°C with 5% CO_2_, and the cell viability was calculated by detecting the OD_450_-nm value with an enzyme marker. The same assay was used to detect cell viability when AA (0.01, 0.05, 0.1, 0.5, 1, and 5 μg/mL) was induced alone and co-induced with the 10 μg/mL of LTA for 12 h.

### Quantitative polymerase chain reaction analysis

Primers were designed for the above-mentioned genes using Primer Premier 6.0 software based on the corresponding sequences of messenger RNA (mRNA) for *IL-1*β, *IL-2, IL-6*, and β*-Actin* in cows provided in the National Center for Biotechnology Information database (Table 1). Reactions were carried out using a Light Cycler^®^ 96 PCR system under the following thermal cycling conditions: A 300-s initial denaturation phase at 95°C, followed by 45 cycles of 95°C for 15 s and 60°C for 30 s. All tests were carried out in triplicate. β*-Actin* was utilized as a reference gene.

**Table 1:** Primers used in a polymerase chain reaction (PCR) for amplification.

**Gene**	**Accession**	**Sequences (5' → 3')**	**Product**
	**No**.		**length (bp)**
*β-actin*	AY141970.1	F: CAACCGTGAGAAGATGACCCA	293
		R: TGTCACGGACGATTTCCGCTC	
*IL-1β*	NM_174093.1	F: TCCGACGAGTTTCTGTGTGA	206
		R: ATACCCAAGGCCACAGGAAT	
*IL-2*	NM_180997.2	F: GCCCAAGGTTAACGCTACAG	106
		R: GGGGTTCAGGTTTTTGCTTGG	
*IL-6*	NM_173923.2	F: TCCTGAAGCAAAAGATCGCA	221
		R: CTGACCAGAGGAGGGAATGC	
		R: CCAAAGTAGACCTGCCCAGA	

### Sample collection

MAC-T cells were planted in 36 10-cm^2^ culture dishes at a density of 1 × 10^7^ cells per dish. They were treated with LTA for 24 h to constitute the LTA group and with AA for 12 h to constitute the AA group. The cells were pretreated with LTA for 24 h followed by treatment with AA for 12 h to constitute the LTA + AA group. Each of the three groups mentioned above, and the control group, were designed with nine repetitions. The 36 cell samples were divided equally into three portions, each containing these four groups (three replicates each). Proteomic and metabolomic analyses were performed on 12 cell samples. The cell-culture solution was discarded, and the cells were washed with PBS and treated with trypsin. The medium was added to terminate the digestion and the medium aspirated. The cells were washed with PBS and stored at −80°C. Transcriptome analysis was conducted on another 12 cell samples. These cells were washed quickly with PBS; 1 mL Trizol was added to each culture dish, followed by incubation on ice for 2–3 min. After blowing, they were transferred to 2-mL RNase-free centrifuge tubes and stored at −80°C.

### Proteome analysis

The nano-ultra-performance liquid chromatography (UPLC) (EASYnLC1200) was paired to Q Exactive HF-X equipment (ThermoFisher Scientific) with the nanoelectrospray ion source isolated and analyzed 2 μg of total peptides for each sample. A reversed-phase column (100 μ ID × 15 cm, Reprosil Pur 120 C18 AQ, 1.9 μ, Dr. Maisch) was used for separation. Mobile phases were phase A (H_2_O with 0.1% FA, 2% ACN) and phase B (80% ACN, 0.1% FA). The material was separated using a 90-min gradient at 300 nL/min. Gradient B: 2–5% for 2 min, 5–22% for 68 min, 22–45% for 16 min, 45–95% for 2 min, and 95% for 2 min.

Data-dependent acquisition (DDA) was carried out using an Orbitrap analyzer in profile and positive mode at a resolution of 120,000 (@200 m/z) and m/z range of 350–1,600 for mass spectrometry (MS)1; the resolution was adjusted to 45 k with a fixed initial mass of 110 m/z for MS2. The AGC (automatic gain control) objective for MS1 was set at 3E6 with a maximum IT of 30 ms and MS2 was set to 1E5 with a maximum IT of 96 ms. High-energy collision dissociation fragmented the top 20 most energetic ions with normalized collision energy (NCE) of 32% and an isolation window of 0.7 m/z. Peaks with a single charge >6 were eliminated using the DDA method, which included a dynamic exclusion time frame of 45 s.

The integrated SEQUEST HT search engine and proteome discoverer software version 2.4.0.305 were used for processing the files (vendor's raw MS). The lists of MS spectra were compared to UniProt FASTA databases at the genus and species levels (Bos taurus 9913-2021-9. fasta), with the fixed modifications including tandem mass tag (TMT) Pro (N-term) and TMT Pro (K) and variable modifications including acetyl (protein N-term) and oxidation (M). The trypsin was utilized to deal with two missed cleavage(s). The false discovery rate at the peptide-to-spectrum match and peptide levels was adjusted to 0.01. An initial precursor mass fluctuation of 10 ppm and a fragment mass bias of 0.02 Da were selected for peptide identification. Proteins were quantified using unique peptides and razor peptides and normalized to total peptide amounts. All other options were set to default values.

### Transcriptome analysis

Following the total RNA extraction with Trizol reagent, the quantity and purity of total RNA were determined by Bioanalyzer 2100 and RNA 6000 Nano LabChip Kit (Agilent, CA, USA, 5067-1511), and further, high-quality RNA samples (RNA Integrity Number >7) were selected to construct a sequencing library. mRNA was isolated from total RNA (5 μg) using Dynabeads Oligo (dT) (Thermo Fisher, CA, USA) and purified twice. Following purification, mRNA was fragmented with the Magnesium RNA Fragmentation Module (NEB, cat.e6150, USA), using divalent cations at 94°C for 5–7 min. The cleaved RNA fragments were reverse transcribed into complementary DNA (cDNA) using SuperScriptTM II Reverse Transcriptase (Invitrogen, cat.1896649, USA), followed by *E. coli* DNA polymerase I (NEB, cat.m0209, USA), RNase H (NEB, cat. m0297, USA), and deoxyuridine triphosphate solution (Thermo Fisher, cat. R0133, USA). In preparation for ligation to index adapters, the blunt ends of each strand needed to be treated with A bases. Accurate ligation was possible since each adapter had a T-base overhang for ligation to the A-tailed DNA. After the fragments were ligated to the double-indexed adapters and size-selected using AMPureXP beads, the ligated products were denatured at 95°C for 3 min, followed by eight cycles of denaturation at 98°C for 15 s, annealing at 60°C for 15 s, extension at 72°C for 30 s, and further PCR amplification with thermolabile uracil-DNA glycosylase enzyme (NEB, cat.m0280, USA) at 72°C for 5 min. The resulting cDNA library had an average insert size of 300 ± 50 bp. Finally, 2,150 bp paired-end sequencing (PE150) was performed using an Illumina NovaseqTM 6000.

Raw reads generated from RNA-seq were subjected to strict quality control (Trimmomatic version 0.39) and further aligned to Bos taurus ARS-UCD1.2 using STAR version 2.7.9a with an average mapping rate over 90%. Reads aligned to the reference genome were quantified using feature counts in the subread version 2.0.2 package, and further, transcripts per million normalizations were performed using StringTie version 2.1.5. The following analyses were done using the R program. Differentially expressed genes (DEGs) were identified with EdgeR, followed by the Gene Ontology (GO) and Kyoto Encyclopedia of Genes and Genomes (KEGG) pathway enrichment analyses using the online website KOBAS (kobas.cbi.pku.edu.cn/kobas3).

### Metabolome analysis

Samples were treated with 1,000 L of a combined extraction solution containing an isotope-labeled internal standard (acetonitrile: methanol: water = 2:2:1). Treated samples were vortexed for 30 s and then frozen and thawed thrice using liquid nitrogen. The samples were further sonicated in an ice-water bath for 10 min and incubated at −40°C for 1 h. For liquid chromatography (LC)/MS analysis, the supernatant was also extracted after centrifugation of the sample at 12,000 × *g* for 15 min at 4°C. Both extracted supernatants were used to prepare quality control samples.

LC-MS/MS analysis (Orbitrap MS, Thermo) was performed using an ultra-high-performance (UHP)LC system (Vanquish, Thermo Fisher Scientific) capable of connecting to a UPLC BEH amide column (2.1 mm 100 mm, 1.7 m) of a Q Exactive HFX mass spectrometer. The mobile phase used in the experiments consisted of 25 mmol/L ammonium acetate and 25 mmol/L aqueous ammonium hydroxide (pH = 9.75) (A) and acetonitrile (B); the temperature of the autosampler was set to 4°C; the required experimental sample volume was 3 L.

The mass spectrometer used in the experiment performed MS/MS spectrum acquisition using the information data acquisition (IDA) mode (Xcalibur, Thermo) in the acquisition software. In IDA mode, the acquisition program was continuously evaluated, and the complete MS spectrum was finally scanned. The electrospray ionization source conditions used were as follows: Sheath and auxiliary gas flow rates of 30 and 25 Arb, respectively, capillary temperature of 350°C, full MS resolution and MS/MS resolution of 60,000 and 7,500, respectively, collision energy 10/30/60 in NCE mode, and spray voltage set to 3.6 kV (positive) or −3.2 kV (negative). The raw data were converted to mzXML format by ProteoWizard and processed using XCMS-based in-house software (created in R), causing peak identification, extraction, alignment, and integration. An internal MS2 database was used for metabolite annotation with an annotation cutoff set at 0.3.

### Western blot

Western blotting experiments were done to verify differential expression. Equal amounts of proteins from the control, LTA, AA, and LTA + AA groups were separated by gel electrophoresis and transferred to polyvinylidene difluoride membranes. Membranes were further blocked for 1 h at room temperature with 5% nonfat dried milk in Tris-buffered saline (TBS) containing 0.1% Tween 20 before being probed with antibodies against IL-1β (1:500, ab205924, Abcam, USA), IL-2 (1:500, bs4586M, Bioss, Beijing, China), IL-6 (1:200, bs4587M, BIOSS, Wuhan, China), IL-8 (1:500, bs0780R, Bioss, Beijing, China), TNF-α (1:500, AF704, Affinity, USA), and β-actin (1:5000, bsm-33036M, BIOSS, Beijing, China). These antibodies responded to homologous epitopes observed in samples. Membranes were washed thrice with prepared TBS solution containing 0.1% Tween 20 and labeled with goat anti-rabbit (1:5,000, S0001, Affinity, USA) and goat anti-mouse (1:5,000, BA1050, Boster, China) immunoglobulin G conjugated to horseradish peroxidase, washed thrice with PBS containing 0.1% Tween 20, and visualized using the WesternBright ECL.

### Statistical analysis

All experiments were repeated at least three times. The data were organized in SPSS, and graphs were plotted using GraphPad Prism 9 packages. The results of qRT-PCR and CCK-8 were expressed as means ± S.E.M. SPSS version 26.0 was used to analyze all the data, and significant differences (*P* < 0.05) were detected by Duncan's multiple-range test in one-way analysis of variance. The results of Western blot were statistically analyzed with ImageJ V1.8.0 and IBM SPSS version 26.0. The results were expressed as means ± S.E.M. SPSS software version 26.0 was used to analyze all the data, and significant differences (*P* < 0.05) were detected by the Duncan's multiple-range test in two-way analysis of variance.

## Results

### Establishment of cell model

**Figure 1:**
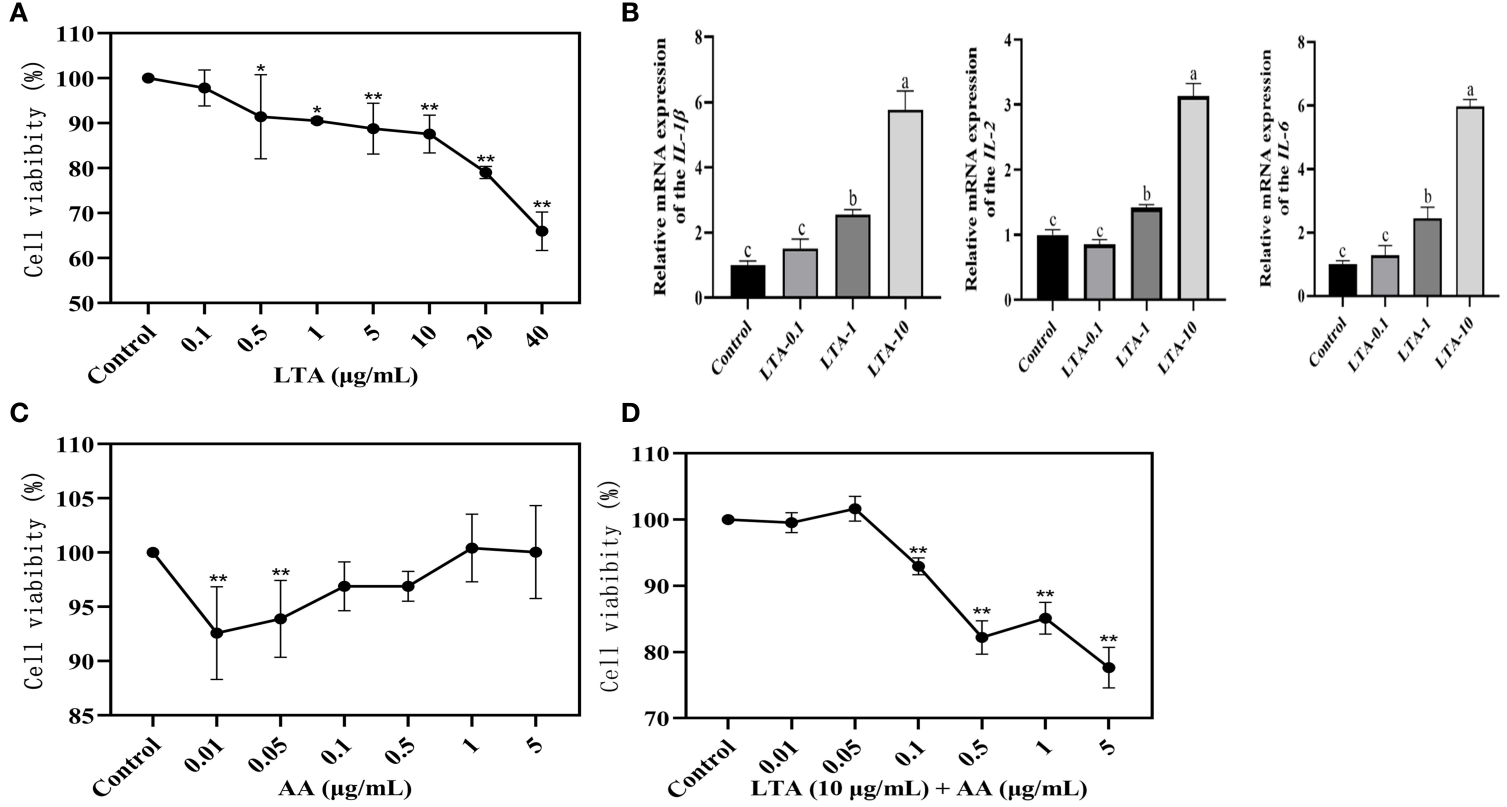
**(A)** Results of Cell Counting Kit (CCK)-8 assay after 24-h induction of bovine mammary epithelial (MAC-T) cells with different concentrations of lipoteichoic acid (LTA). **(B)** Quantitative polymerase chain reaction (qPCR) of LTA-induced cells. LTA-0.1, LTA-1, and LTA-10 represent LTA-induced cell concentrations of 0.1, 1, and 10 μg/mL. **(C)** Results of CCK-8 assay after 12-h induction of MAC-T cells with different concentrations of arachidonic acid (AA). **(D)** Results of CCK-8 assay after 36-h induction of MAC-T cells with different concentrations of AA. The mean value ± SD from three independent experiments was used to analyze the relative transcript levels for each time point using the Duncan's multiple range test. *Indicates significant differences between the control group and the LTA (0.1 or 0.5 μg/mL) groups (*P* < 0.05), ** indicates significant differences between the control group and the LTA (5, 10, 20, or 40 μg/mL) groups, the control group and the AA (0.01 or 0.05 μg/mL) groups, and the control group and the LTA (10 μg/mL) + AA (0.1, 0.5, 1, or 5 μg/mL) groups (*P* < 0.01). “a” indicates significant differences between the control group and the LTA (10 μg/mL) group (*P* < 0.01); “ b” indicates significant differences between the control group and the LTA (1 μg/mL) group (*P* < 0.05); “c” indicates not significant differences between the control group and the LTA(0.1 μg/mL) group.

The cellular viability of LTA at different concentrations (0.1, 1, 5, 10, 20, or 40 μg/mL) after 24 h of action on MAC-T was assessed by the CCK-8 assay. The effect of LTA on cell viability was dose-dependent, with a significant decrease in MAC-T cell activity with increasing LTA concentration (*P* < *0.05*), and subsequent experiments required 0.1, 1, and 10 μg/mL of LTA to induce MAC-T (Figure 1A). The gene expressions of *IL-1*β, *IL-2*, and *IL-6* were highest when the LTA concentration was 10 μg/mL (Figure 1B) (*P* < 0.05), and the cell viability was close to 90%, therefore, it was selected as the optimal inducer concentration of LTA. To screen the inducer concentration of AA, it was necessary to ensure high cell viability both when induced alone and when co-induced with LTA. We observed that the cell viability of AA inducer concentration of 0.1 μg/mL was >95% when induced alone, and it was significantly higher than 0.01 and 0.05 μg/mL (Figure 1C) (*P* < 0.05). After adding LTA, the cell viability of 0.1 μg/mL was also close to 95%, which was significantly higher than 0.5, 1, and 5 μg/mL (Figure 1D) (*P* < 0.05). Based on this, we selected 0.1 μg/mL as the optimal concentration of AA for the subsequent experiments.

### Quality evaluation of proteome and differentially expressed proteins analysis

**Figure 2:**
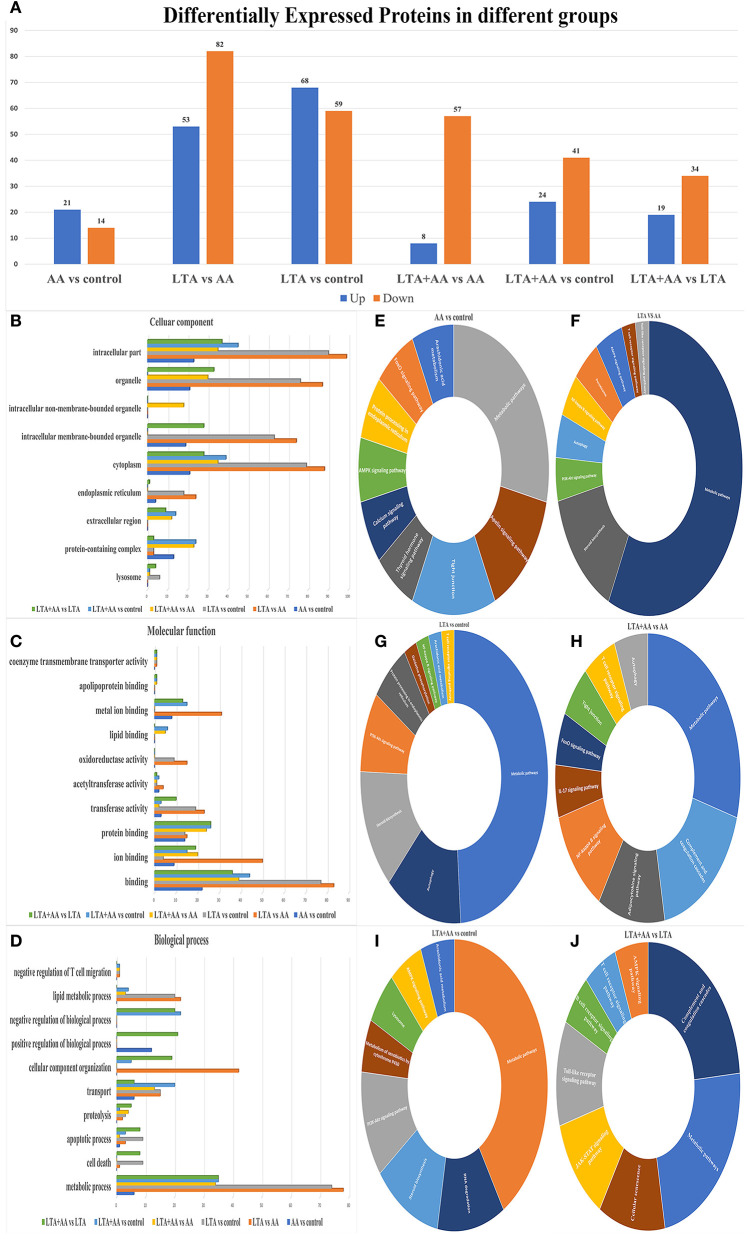
**(A)** Differentially expressed proteins (DEPs) in different groups. **(B–D)** Gene ontology analysis of DEPs. Proteins were annotated by biological processes (BP), cellular components (CC), and molecular function (MF). **(E–J)** KEGG functional analysis of DEPs.

Forty-four thousand fifty unique peptides were identified in 6,780 protein samples after analyzing MS/MS spectra with the Mascot program. Four hundred seventy-nine proteins were identified to be differently expressed in samples, with 192 being upregulated and 287 being downregulated, using fold change >1.2 and *P*-value <0.05 (Figure 2A). The proteins were annotated by GO analysis to be involved in CC (cellular component), MF (molecular function), and BP (biological process). DEPs in the LTA vs. AA group were the most enriched in intracellular part (99 DEPs), organelle, intracellular membrane-bounded organelle (74 DEPs), cytoplasm (88 DEPs), and endoplasmic reticulum (24 DEPs) from CC (Figure 2B), metal ion binding (31 DEPs), oxidoreductase activity (15 DEPs), acetyltransferase activity (4 DEPs), transferase activity (22 DEPs), ion binding (50 DEPs), binding (83 DEPs) from MF and lipid metabolic process (22 DEPs), CC organization (42 DEPs), and metabolic process (78 DEPs) in MF (Figure 2C), indicating that LTA and AA have opposing regulatory functions in certain metabolism and redox pathways. DEPs in the LTA vs. control group were the most enriched in the lysosome (9 DEPs) from CC, apoptotic process (9 DEPs), and cell death (9 DEPs) in BP (Figure 2D), indicating that LTA mainly acts on host cell apoptosis and autophagy pathways. It is noteworthy that DEPs in the AA vs. control group were not enriched in lysosomes from CC, oxidoreductase activity in MF, and negative regulation of T-cell migration, lipid metabolic process, and cell death in BP. DEPs in the LTA + AA vs. LTA group were not enriched in negative regulation of T-cell migration and lipid metabolic process. Simultaneously, only the DEPs of AA vs. control groups and LTA + AA vs. LTA groups were enriched in positive regulation of BPs (12 and 21 DEPs, respectively). This indicates that AA rarely affects lipid metabolism and immune response pathways in direct host cells, albeit can affect LTA-induced host cells, thereby, reducing the effect of LTA on cells.

The DEPs were further evaluated using the KEGG pathway analysis (Figures 2E–J). The DEP enrichment pathways involved in each group were screened, and some immune and metabolism-related pathways were selected for display. For example, the most enriched DEPs in each group were metabolic pathways. DEPs in AA vs. control, LTA vs. control, and LTA + AA vs. control were involved in the AA metabolism pathway. Remarkably, immune pathways, such as Nuclear Factor-Kappa B (NF-κB) signaling pathway, autophagy, T-cell receptor signaling pathway, adenosine monophosphate-activated protein kinase (AMPK) signaling pathway, toll-like receptor signaling pathway, etc., were upregulated or downregulated to various extents.

### Quality evaluation of the transcriptome and differentially expressed genes analysis

**Figure 3:**
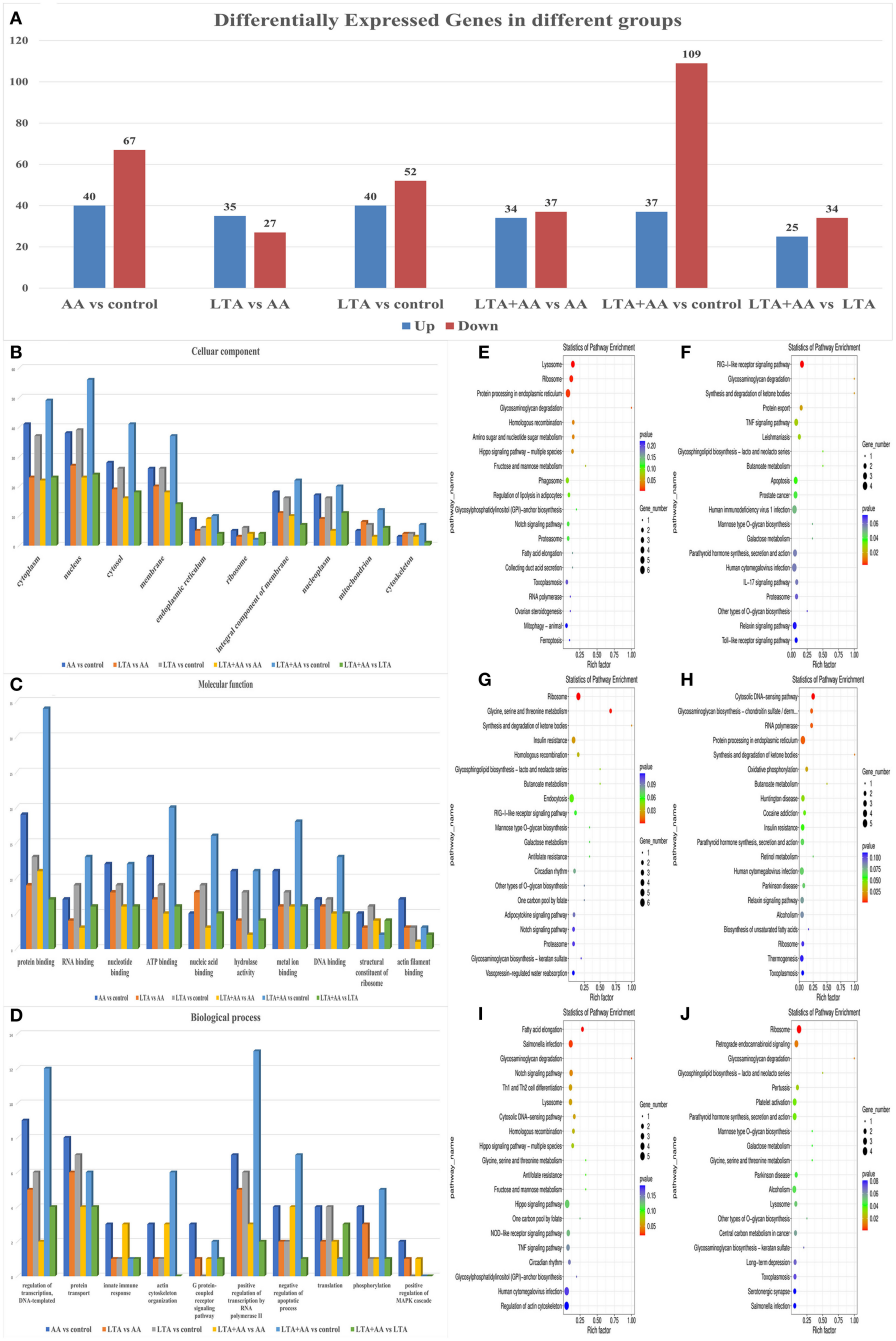
**(A)** Differentially expressed genes (DEGs) in different groups. **(B–D)** Gene ontology analysis of DEGs. Proteins were annotated by biological processes (BP), cellular components (CC), and molecular function (MF). **(E–J)** KEGG functional analysis of DEGs.

Following statistical analysis and quantile normalization, 537 DEGs (*P* < *0.05*, fold change >2) were observed, with 211 being upregulated and 326 being downregulated (Figure 3A). GO annotation and KEGG pathway enrichment studies were used to investigate the biological roles of the DEGs. GO annotation in the CC category of most DEGs was primarily involved in the cytoplasm, nucleus, cytosol, and membrane (Figure 3B). A substantial number of DEGs were observed to be involved in protein binding, nucleotide binding, ATP binding, hydrolase activity, and metal ion binding in the molecular function category (Figure 3C). The GO annotation of the DEGs was predominantly engaged in transcription control, DNA-templated, protein transport, and positive transcription regulation by RNA polymerase II in the BP category (Figure 3D). It is noteworthy that the AA vs. control group had the highest number of differential gene enrichment among the other groups in the G protein-coupled receptor signaling pathway, innate immune response, and positive regulation of mitogen-activated protein kinase (MAPK) cascade, indicating that AA can participate in these pathways to affect cellular immunity. The KEGG enrichment analysis was performed on DEGs, and the top 20 KEGG pathways with the smallest *P*-value in each group were selected. Remarkably, some of these metabolic and immune pathways had substantial DEGs enrichment, such as lysosome, fatty acid elongation, IL-17 signaling pathway, toll-like receptor signaling pathway, etc. (Figures 3E–J).

### Quality evaluation of the metabolomics and differentially expressed metabolites analysis

**Figure 4:**
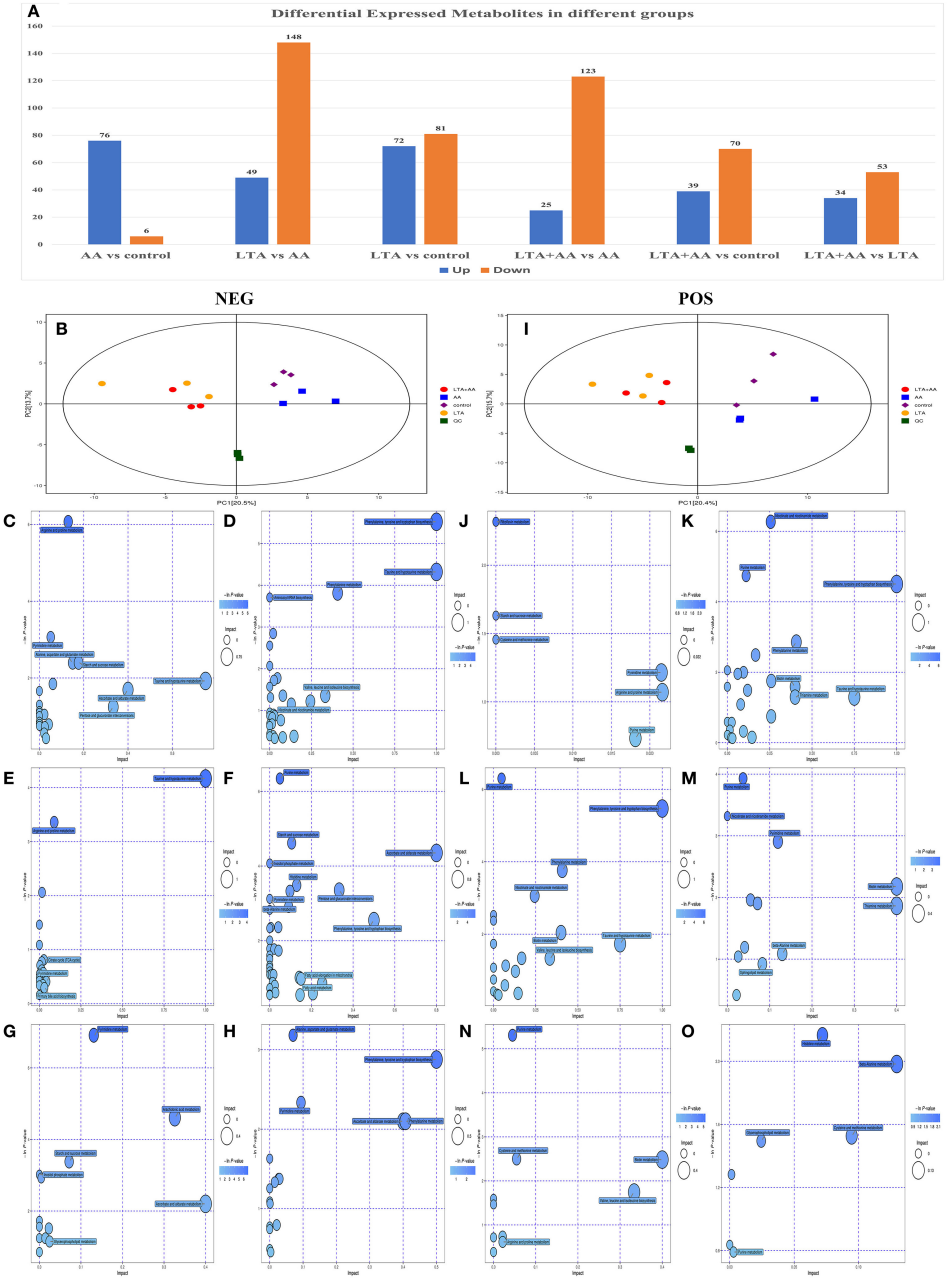
**(A)** Differentially expressed metabolites in different groups. **(B)** Score scatter plot for principal component analysis (PCA) model TOTAL with Quality Control (QC) in negative ion mode (NEG). **(I)** Score scatter plot for PCA model TOTAL with QC in positive ion mode (POS). **(C–H)** Kyoto Encyclopedia of Genes and Genomes (KEGG) functional analysis of differentially expressed metabolites in NEG. **(J–O)** KEGG functional analysis of differentially expressed metabolites in POD.

Following quantile normalization and statistical analysis, 776 different metabolites (*P* < *0.05*, fold change >2) were identified, of which 246 were upregulated and 530 were downregulated (Figure 4A), and separated clusters were observed in the metabolomic profiles between the four groups through the negative ion mode (NEG)- and positive ion mode (POS)-principal component analysis score map (Figures 4B,I). The results of the metabolic pathway analysis of differential metabolites were obtained through metabolite databases, such as KEGG and PubChem (Figures 4C–H,J–O). Pathway analysis was performed on different metabolites, and it is noteworthy that some pathways were significantly enriched, such as arginine and proline metabolism pathway, phenylalanine, tyrosine, and tryptophan biosynthesis pathway, riboflavin metabolism pathway, nicotinate and nicotinamide metabolism pathway, ascorbate and aldarate metabolism pathway, and glycerophospholipid metabolism pathway. This result suggests that both LTA and AA can affect the synthesis of some key metabolites to regulate cellular metabolism and thus affect cellular immune activity.

### Combined analysis of proteomic, transcriptomic, and metabolomic data

**Figure 5:**
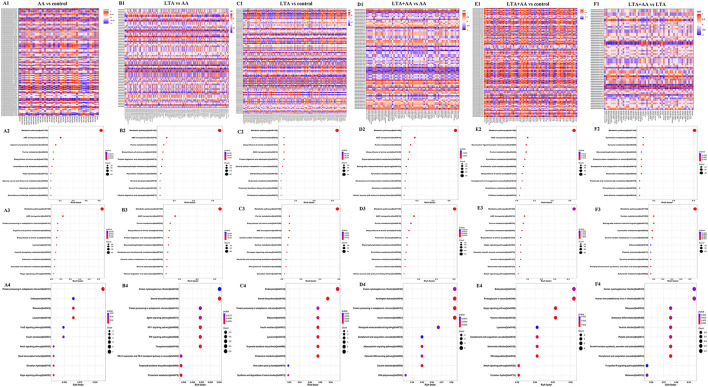
**(A1,B1,C1,D1,E1,F1)** Correlation heat map in red (corr = 1), blue (corr = −1), and white (corr = 0). The correlation of *P* < *0.05* is marked with an “*” in the graph. Vertical coordinates represent differential genes; horizontal coordinates represent differential proteins. **(A2,B2,C2,D2,E2,F2)** Kyoto Encyclopedia of Genes and Genomes (KEGG) functional analysis of the combined results of differential metabolites and differential proteins comparisons. **(A3,B3,C3,D3,E3,F3)** KEGG functional analysis of the combined results of differential metabolites and differential genes comparisons. **(A4,B4,C4,D4,E4,F4)** KEGG functional analysis of the combined results of differential proteins and differential genes comparisons.

To determine the complementarity and integration of mRNAs and proteins, we analyzed the interaction correlation between the DEGs and DEPs based on the Pearson's correlation coefficients (Figures 5A1–F1). We used the KEGG pathway analysis to identify genes and metabolomics with changes at multiple levels by combining data from proteomics, transcriptomics, and metabolomics. Gene expression and metabolomic results were analyzed by integrating the KEGG pathway, and differential variable enrichment results were analyzed. For example, in the KEGG association analysis of metabolome, transcriptome, and proteome, it can be observed that every other group involves ABC transporters, biosynthesis of amino acids, and purine metabolism, except for the LTA + AA vs. LTA group (Figures 5F2,F3). The comparison of AA vs. control (Figures 5A2,A3), LTA vs. control (Figures 5C2,C3), and LTA + AA vs. control (Figures 5E2,E3) groups revealed that AA affected metabolism pathway and Hippo signaling pathway. However, when LTA alone induced cells, changes in protein digestion and absorption and the steroid biosynthesis pathway were observed. The results of the KEGG enrichment analysis of transcriptome and proteome revealed that the regulatory immune and metabolic pathways varied in different groups. For example, AA regulated the forkhead box protein O1 (FoxO) signaling pathway and lysosome pathway in the AA vs. control group (Figure 5A4). LTA alone induced cells to mainly regulate the TNF signaling pathway and steroid biosynthesis (Figure 5C4). Except for LTA + AA vs. LTA (Figure 5F4) and LTA + AA vs. control (Figure 5E4), the other four groups were involved in protein processing in the endoplasmic reticulum pathway (Figures 5B2–B4, D2–D4). This indicates that LTA and AA play opposite regulatory roles in this pathway.

### Verification of proteome and transcriptome

**Figure 6:**
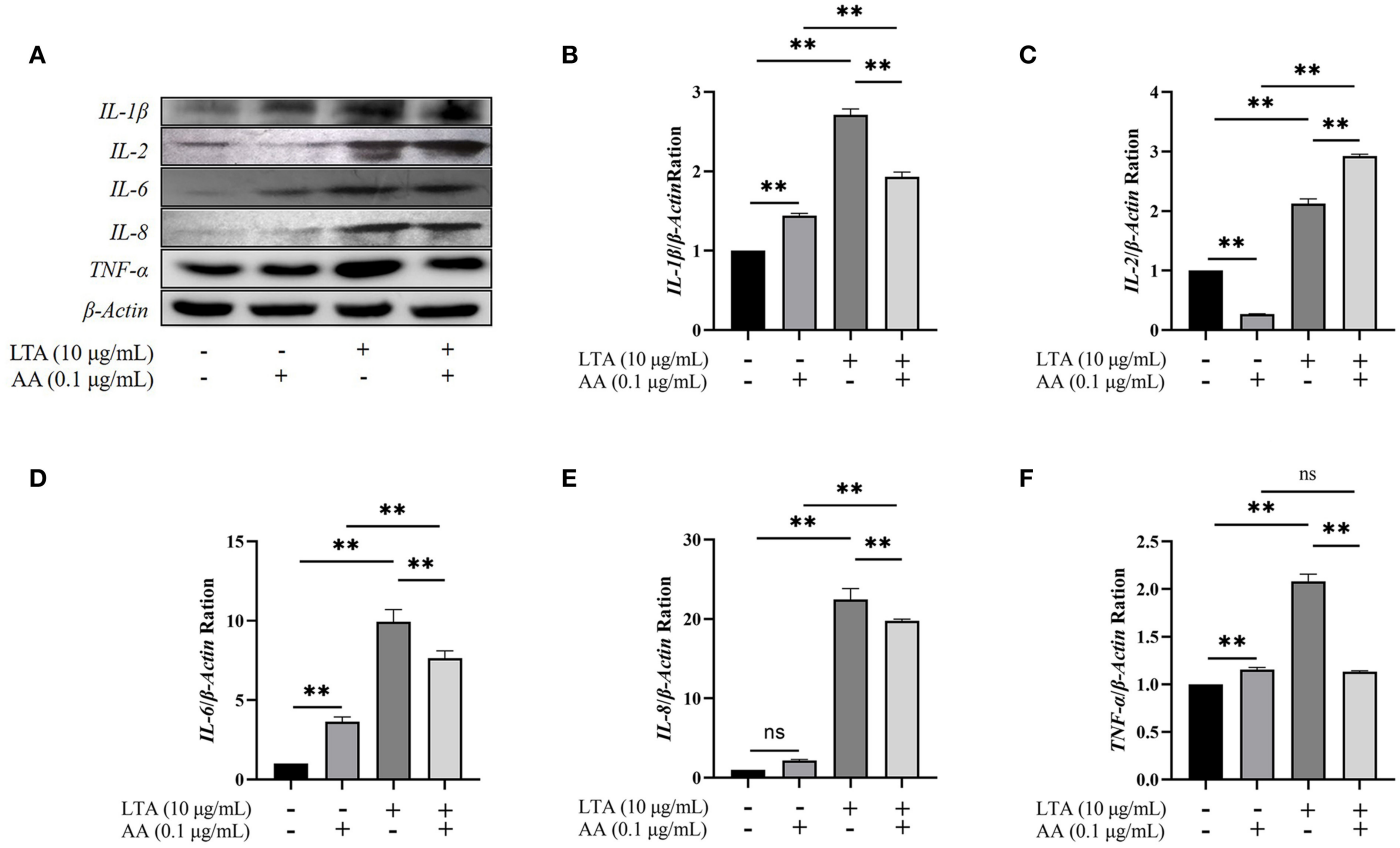
**(A)** Western blot of *interleukin* (*IL*)*-1*β, *IL*−*2, IL*-6, *IL*-8, and *tumor necrosis factor* (*TNF*)-α among groups. **(B–F)** The quantification histogram of *IL-1*β, *IL-2, IL-6, IL-8*, and *TNF*-α protein expression normalized by β-Actin. “–” represents cells without LTA or AA; “+” represents cells containing lipoteichoic acid (LTA) or arachidonic acid (AA). The data are expressed as mean ± SD from three independent experiments. The *P*-value was calculated by two-way analysis of variance (ANOVA) for paired samples. **Indicates significant differences between the AA group and LTA + AA group, LTA group and LTA + AA group, the control group and the LTA group, and the control group and the AA group (*P* < 0.01). “ns” indicates not significant differences between the control group and the AA group (*IL-8* protein expression), and the AA group and the LTA + AA group (*TNF*-α protein expression).

Western blot demonstrated that *IL*-1β, *IL*-6, *IL*-8, and *TNF*-α were upregulated in AA, LTA, and LTA + AA groups whereas IL-2 was downregulated in the AA group and upregulated in other groups, consistent with and highlighting the reliability of proteomic and transcriptomic data (Figure 6).

## Discussion

*S. aureus* is the main pathogenic agent of mastitis in dairy cows and is more difficult to prevent and cure than other pathogens, owing to its antibiotic resistance and strong environmental adaptability. LTA is the main pathogenic factor of *S. aureus*. Studies on the effect of AA on the immune and metabolic regulation of the body have been receiving great attention. In this experiment, we demonstrated the cellular regulatory changes by establishing a model of LTA-induced inflammation and treating it with exogenous AA.

The results of the proteomic and metabolomic comparative analysis revealed that LTA induction in host cells induced not only the upregulation of a large number of metabolic regulatory proteins (Table 2) but also the downregulation of some specific metabolic pathways, such as *cytochrome P450 family 2 subfamily C19* protein in the AA metabolic pathway, which plays an important role mainly as a cytochrome P450, which is a derivative of amino acid, in drug metabolism, bioactivation, and inactivation of xenobiotics (17). The transcriptomic data also revealed an upregulation of *Actin Related protein 2/3 complex* (*ARPC*)*3* and *ARPC4* expression, which are mainly involved in the bacterial invasion of epithelial cells pathway and affect actin synthesis. They play an important role in the invasion of host cells by *E. coli*; however, no studies have been reported on *S. aureus* (18). These two genes and four other down-regulated genes, *Sorting Nexin 5* (*SNX5*), *Charged Multivesicular Body Protein* (*CHMP*)*3, RAB4A*, and *RAB5A*, are involved in the cellular endocytosis pathway, mainly affecting clathrin-coated vesicle, primary endosome, and secondary endosome formation (19–22). It is noteworthy that the upregulation of the expression of the two genes, *protein kinase cGMP-dependent* (*PRKAG*)*1* and *NF-*κ*B Inhibitor Alpha* (*NFKBIA*), causes apoptosis (23, 24).

**Table 2:** Upregulated metabolic regulatory proteins in lipoteichoic acid (LTA) vs. control group.

**Protein**	**Gene**	**Peptide**	**Fold change**	**Accession**
RRM2 protein	*RRM2*	7	1.244	Q2HJE7
3-hydroxy-3-methylglutaryl coenzyme A synthase	*HMGCS1*	8	1.549	Q3ZC79
Delta(24)-sterol reductase	*DHCR24*	6	1.301	A0A3Q1M7E0
Lanosterol 14-alpha demethylase	*CYP51A1*	5	1.288	Q4PJW3
Squalene synthase	*FDFT1*	5	2.469	Q32KR6
Exo-alpha-sialidase	*NEU1*	4	1.214	A0A3Q1MPM1
Acetyl-CoA acetyltransferase	*ACAT2*	2	1.211	Q17QI3
Thymidylate synthase	*TYMS*	3	1.303	F1MY63
Heme oxygenase 1	*HMOX1*	1	1.314	Q5E9F2
Protein-tyrosine-phosphatase	*MTMR4*	1	1.207	A0A3Q1N088
Isopentenyl-diphosphate Delta-isomerase 1	*IDI1*	2	1.439	Q1LZ95
Branched-chain-amino-acid aminotransferase	*BCAT2*	1	1.220	Q0V8J6
Corrinoid adenosyltransferase	*MMAB*	1	1.298	E1B6Z7
Squalene monooxygenase	*SQLE*	1	1.518	A5D9A8
Fatty acid desaturase 1	*FADS1*	1	1.230	F1N4L8
Glutathione transferase	*GSTA4*	1	1.361	A0A3Q1M0K8
7-dehydrocholesterol reductase	*DHCR7*	1	1.231	Q5E9J5
Polypeptide N-acetylgalactosaminyltransferase	*GALNT16*	1	1.234	A6QLD9
Sterol-C5-desaturase	*SC5D*	1	1.241	Q3SYX8
Diphosphomevalonate decarboxylase	*MVD*	1	1.394	Q0P570

In contrast, we observed that when induced alone, AA modulated some proteins and gene expression to enhance cellular activity in cells. First, its effect on metabolic regulation was significantly lower than that of LTA, which mainly decreased *prostaglandin E synthetase* (*PTGES*)*3* expression, thus, affecting the AA metabolic pathway. Notably, the phagocytic activity of macrophages induced by LPS was enhanced by the reduced *PTGES3* expression level (25). Second, during protein processing in the endoplasmic reticulum, expression levels of *Translocation Associated Membrane Protein* (*TRAM*)*1, SEC61 Translocon Subunit Gamma* (*SEC61G*), and *Signal Sequence Receptor Subunit 1* (*SSR1*) are upregulated, causing high expression of the *DnaJ Homolog Subfamily Member B1* (*DNAJB1*) gene and promoting *Heat Shock Protein* (*HSP*)*40* synthesis. *HSP40* plays a critical role in the regulation of mammalian cell proliferation, survival, and apoptosis (26). *HSP40* and *HSP70* proteins are inextricably linked, and studies have reported that *HSP40* proteins can regulate the ATPase activity of *HSP70* proteins whereas the *HSP70* family can reduce apoptosis by inhibiting the activation pathway of the stress enzymes (27). Last, we observed that expression levels of *Laminin Gamma 1*(*LAMC1*), *Insulin Receptor* (*INSR*), *Filamin B* (*FLNB*), and *Casein Kinase 1 Epsilon* (*CSNK1E*) were downregulated and *Ribosomal Protein S6 Kinase A1* (*RPS6KA1*) expression was upregulated in the MAPK, FoxO, and phosphatidylinositol 3-kinase (PI3K)-protein kinase B (Akt) signaling pathways, and all these changes in gene expression reduced apoptosis (28–32). Additionally, *Vacuolar Protein Sorting 25 Homolog* (*VPS25*), *Charged Multivesicular Body Protein* (*CHMP*)*3*, and *Capping Actin Protein Of Muscle Z-Line Subunit Beta* (*CAPZB*) expressions were upregulated in the endocytosis pathway, which is involved in metabolism, and these genes promote endosome formation, thus, increasing cellular metabolism and transduction of signaling substances (20, 33, 34).

**Figure 7:**
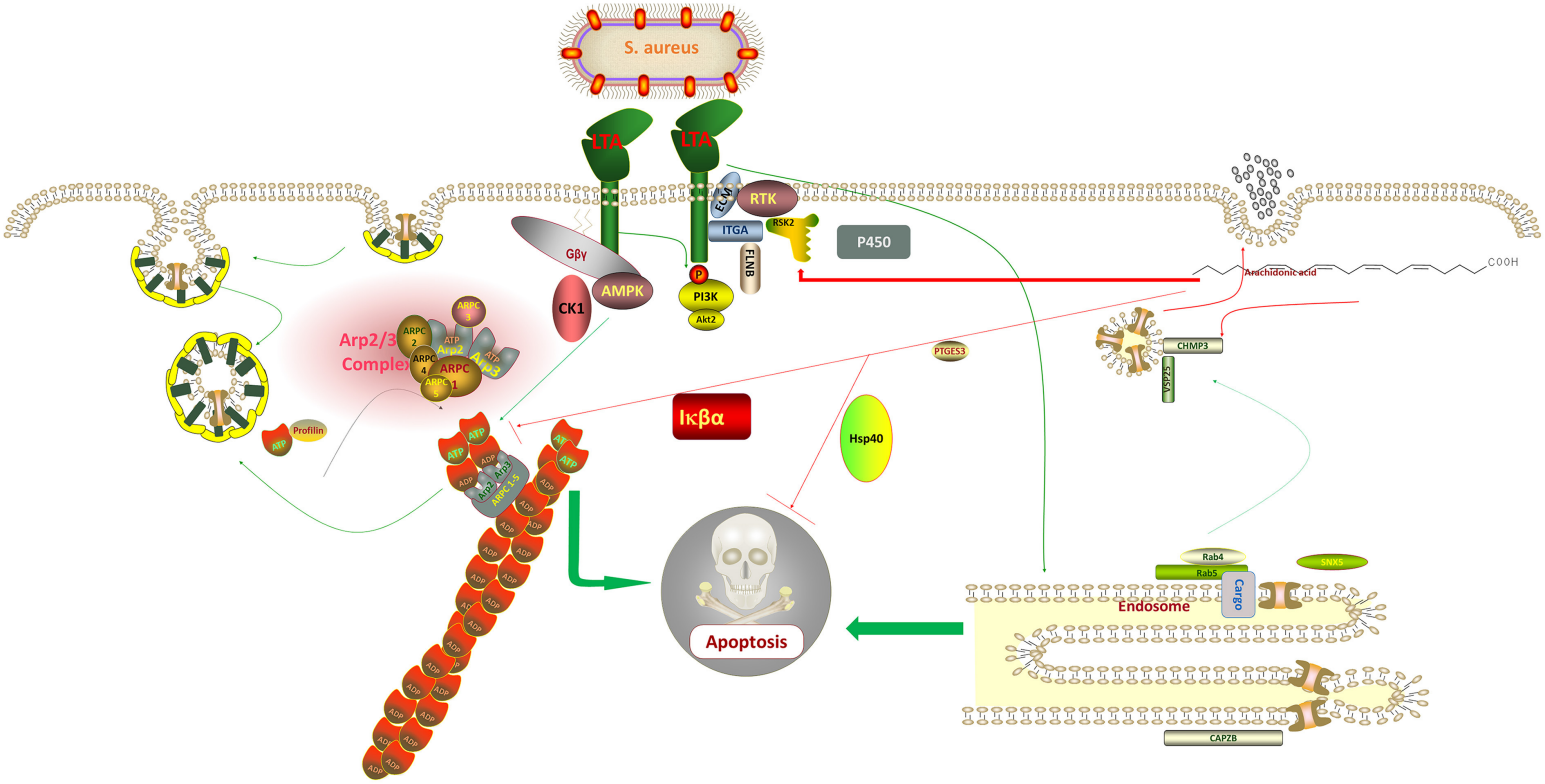
*Staphylococcus aureus* regulates the expression of adenosine monophosphate-activated protein kinase (AMPK), protein kinase B (Akt), and Actin Related protein 2/3 complex (ARP2/3) through lipoteichoic acid (LTA), which affects the metabolic activity of host cells and promotes the uptake of its metabolites, thus, increasing its division and replication capacity and eventually causing apoptosis. In contrast, arachidonic acid (AA) inversely regulates the synthesis of these proteins, thereby, protecting the normal diversion of metabolites from the host cell and ultimately inhibiting apoptosis.

When exogenous AA was added to the host cells following LTA induction, the expressions of *ARPC3* and *ARPC4* in the pathway of bacterial invasion of epithelial cells were observed to be significantly downregulated when compared with LTA alone, indicating that AA can protect the host cells by regulating their expression and reducing the invasion efficiency of *S*. *aureus*. The upregulation of *CHMP3* expression in the endocytosis pathway is a noteworthy point, and some studies have reported that it can promote the invasion of breast cancer cells by inhibiting *CHMP3* expression (35). The experimental results indicated that AA can defend against pathogenic invasion by regulating the expression of *CHMP3* (Figure 7). The outcomes supported our conclusion in the other three data sets where these three genes were expressed. In the LTA + AA vs. control group, *ARPC4* and *CHMP3* expressions were upregulated whereas *ARPC3* expression was unmodified. In the LTA vs. AA and LTA + AA vs. AA groups, *ARPC4* and *ARPC3* expressions were upregulated and *CHMP3* expression was downregulated.

Conclusively, the mechanisms of action of *ARPC3* and *ARPC4* in the invasion of cells by gram-negative bacteria have been widely reported, for example, both are involved in the pathogenic *E. coli, Salmonella*, and *Yersinia* infection pathways (36, 37). However, few mechanisms of action have been reported in gram-positive bacteria. It can be tentatively concluded that *S. aureus* can also use LTA to regulate the upregulation of *ARPC3* and *ARPC4* expression to promote the synthesis of a new cytoskeleton, thus, promoting host cell metabolism and increasing its material and energy uptake to enhance its ability to divide and replicate (38). LTA blocks cytochrome P450 and endosome formation, evades host cell defense, and affects the transport of proteins, nucleic acids, lipids, and other substances within the cell (39). It also activates PI3K-Akt, NF-κB, and FoxO signaling pathways, ultimately causing host cell death (Figure 7). Mastitis in cows is caused by various pathogenic microorganisms including gram-negative and gram-positive bacteria, making it difficult to diagnose and treat mastitis in cows. We look forward to further exploring the mechanism of action of *ARPC3* and *ARPC4* expression to develop new rapid diagnostic methods and drug targets against multiple pathogenic invasions. Additionally, AA has a significant effect on the regulation of these two genes following LTA induction in host cells, inhibition of apoptosis, and increase in cell metabolism and immune response capacity by regulating genes related to pathways such as endocytosis. This also provides a new approach to the treatment of mastitis in cows.

## Data availability statement

The datasets presented in this study can be found in online repositories. The names of the repository/repositories and accession number(s) can be found below: SRA: SRR19971007.

## Author contributions

WD: conceptualization, methodology, software, investigation, formal analysis, and writing—original draft. YC: data curation and writing—original draft. QZ: visualization and investigation. XiaZ, PL, HH, and TL: resources and supervision. YH and XD: software and validation. JH and XinZ: visualization and writing—review and editing. YZ: conceptualization, funding acquisition, resources, supervision, and writing—review and editing. All authors contributed to the article and approved the submitted version.

## Funding

This work was supported by Gansu Province Guiding Science and Technology Innovation Special Project (Project No.: GSCXZX-2019), National Natural Science Foundation of China (Project No.: U21A20262), Agricultural category of key research and development projects of Gansu Provincial Department of Science and Technology (Project No.: 18YF1NA074), Gansu Agricultural University talent introduction funds (Project No.: GAU-KYQD-2019-04), Key Laboratory of Animal Reproductive Physiology and Reproductive Regulation of Gansu Province (Project No.: 20JR10RA563), and Ningxia Autonomous Region Key R&D project (Project No.: 2019BBF02027).

## Conflict of interest

The authors declare that the research was conducted in the absence of any commercial or financial relationships that could be construed as a potential conflict of interest.

## Publisher's note

All claims expressed in this article are solely those of the authors and do not necessarily represent those of their affiliated organizations, or those of the publisher, the editors and the reviewers. Any product that may be evaluated in this article, or claim that may be made by its manufacturer, is not guaranteed or endorsed by the publisher.
